# Bisphosphonate combination therapy for non-femoral avascular necrosis

**DOI:** 10.1186/s13018-019-1152-7

**Published:** 2019-04-24

**Authors:** Sanjay Agarwala, Mayank Vijayvargiya

**Affiliations:** Department of Orthopedics, P.D. Hinduja National Hospital and Medical Research Centre, Mumbai, India

**Keywords:** Bisphosphonates, AVN, Osteonecrosis, Avascular necrosis other than femoral head, VAS score, Bone marrow edema

## Abstract

**Background:**

Avascular necrosis at sites other than femoral head (AVNOFH)/Non-Femoral AVN is a rare entity. No standard of treatment still exists for treating early stages of AVNOFH with most of the cases eventually progressing to a late arthritic stage needing surgical intervention. Bisphosphonates have been shown to prevent disease progression, bone collapse, and the requirement for surgery in avascular necrosis of femoral head. The present study is conducted to evaluate the response of bisphosphonates in the non-surgical management of the early stages of AVNOFH.

**Materials and methods:**

Prospectively collected data of 20 patients diagnosed with an early stage of AVNOFH and treated with the combination of oral alendronate 70 mg weekly and intravenous zolendronic acid (ZA) for 1 year, between Jan 2009 to Dec 2015, was evaluated retrospectively. Clinical evaluation was done using the visual analogue scale (VAS), mean analgesic requirement, and range of motion. Radiographs and magnetic resonance imaging (MRI) were taken to classify the stage of AVN, monitor radiological collapse, and evaluate radiological progression and bone marrow edema changes.

**Results:**

In our analysis of 18 patients (2 lost to follow-up), 5 patients had AVN of the humeral head, 4 patients of the talus, 3 of the lunate, and 2 each of the scaphoid, medial tibial plateau, and second metatarsal head. Pain relief with the drop in VAS score was seen at a mean duration of 4.3 weeks (range 3–13 weeks) after the start of therapy. A 50% reduction in mean analgesic requirement was achieved in the first 6 weeks (2-11 weeks). MRI showed complete resolution of BME in 13 patients at 6 months and in 17 patients (94.4%) at 1 year. Radiological collapse was seen in 6 out of 18 patients at a mean follow-up of 35.3 months (range 14–56 months). Only one out of 18 patients enrolled required surgery.

**Conclusion:**

A combination of oral alendronate and intravenous zolendronic acid provides a pragmatic solution to this rare entity of AVNOFH, where no standard treatment exists.

## Background

Avascular necrosis/Osteonecrosis (AVN) is a debilitating disorder affecting bone architecture leading to destruction and collapse causing secondary arthritis. The femoral head is the commonest site of affection followed by the proximal humerus [1]. Other sites include the knee (distal femur and proximal tibia), talus, scaphoid and rarely capitellum, lunate, vertebra, and facial bone [2–5]. A traumatic or non-traumatic condition can cause interruption of the blood supply to the bone. The common non-traumatic causes for AVN include corticosteroid use, alcoholism, SLE, sickle cell disease, and hemoglobinopathies [6–8]. The presentation of AVN depends upon the site of affection and the stage of disease. Pain is the presenting symptom in most of the cases. Joint mobility is well preserved in early stages but gradually deteriorates once the disease progresses to advanced arthritic stage.

The treatment objective in avascular necrosis is to prevent disease progression, prevent the collapse, obtain pain relief, and preserve joint movement. A multitude of treatment options available for non-femoral avascular necrosis/avascular necrosis other than femoral head (AVNOFH) range from conservative, medical, and surgical modalities; however, no standardized protocol exists. Various pharmacotherapies tried in the past for AVN including ilioprost, nifedipine, and hyperbaric oxygen therapy have not shown significant benefits [9]. Therefore, surgical intervention remains the only treatment option for the sequalae of AVNOFH. Surgical options range from arthrodesis to arthroplasty. Although arthrodesis gives good pain relief, it leads to a significant restriction of activities especially in the Asian population. On the other hand, arthroplasty provides good outcome but when performed at a young age will necessitate at least one revision in the future [10]. Further, arthroplasties other than hip and knee are still evolving and are no match to the normal joint. Therefore, there is a need for a treatment which can halt the disease process and prevent progression to a late arthritic stage of AVN, thus obviating the need for the surgery.

Bisphosphonates have shown promising results in the management of AVN of the femoral head. In a prospective trial of 60 patients with AVN of the femoral head (100 cases), the authors have reported that the use of oral alendronate retards progression prevents collapse, improves clinical outcomes, and potentially avoids arthroplasty [11]. Agarwala et al. in their recent retrospective analysis have shown that the combination of oral alendronate and intravenous zolendronic acid is superior than oral alendronate-only therapy in preventing radiological progression and collapse in AVN [9]. The authors have shown that zolendronic acid (ZA) with its higher bioavailability and faster onset of action complements alendronate in the treatment of AVN. This combination therapy was not only found to have added benefits of both oral alendronate (prevent long-term radiological progression) and intravenous zolendronic acid (early pain relief) but was also safe [9].

There is a paucity of literature on the management of AVN of bones other than femoral head. There are mostly case reports published about rare incidences of AVN at places other than the hip but no definitive treatment method has still been established. We have postulated that a combination of intravenous ZA and oral alendronate which have shown good results in AVN of the femoral head could successfully treat AVN of bones other than femoral head as the pathology of the disease is similar. We have evaluated the clinic-radiological outcome of management of AVN other than the femoral head with ZA and alendronate.

## Methods

Prospectively collected data of 20 patients diagnosed with AVN other than femoral head (AVNOFH) and treated with the combination of oral alendronate and intravenous ZA, between Jan 2009 to Dec 2015, was evaluated retrospectively. Institutional review board (IRB) approval was taken (IRB Approval number-1144-17-SAg). Different classification systems for AVN at locations other than the hip define stages I and II as pre-collapse or early stage and stage III and above as post-collapse or late stage [12–17]. Only those patients presenting with an early stage of AVN (Stage I& II) were included and patients presenting in late stages (Stage III and above) were excluded from the study. We have only included cases of atraumatic AVN, while all the cases presenting after trauma/fracture were excluded from the study. A total of 20 patients with AVN other than femoral head (AVNOFH) were treated at our center during the study period, out of which 2 patients were lost to follow-up and hence excluded from the study group. Mean age at presentation was 38.6 years (range 18–66 years) (Table 1). There were 12 male and 6 female patients. Mean follow-up was 35.3 months (range 14–56 months).

**Table 1:** Table showing the demographic details of the patient enrolled in the study

S. No.	Age/sex	BMI	Side of affection	Dominant (D)/non-dominant (ND)	Site of affection	Stage at start of therapy	Last follow-up (months)	Stage at final follow-up
1	64/M	28.8	Right	D	Humeral head	2	45	3
2	50/F	26.5	Left	ND	Humeral head	1	48	2
3	18/M	20.8	Right	D	Humeral head	2	25	3
4	18/M	21.6	Left	ND	Humeral head	2	25	2
5	30/M	19.6	Left	ND	Humeral head	2	14	3
6	47/M	32.4	Right	-	Talus	2	52	2
7	65/F	26.7	Right	-	Talus	2	44	3
8	66/M	35.6	Right	-	Talus	1	56	3
9	50/M	33.2	Left	-	Talus	2	50	2
10	23/M	21.4	Right	D	Scaphoid	2	32	2
11	32/M	31.9	Left	ND	Scaphoid	2	25	2
12	52/F	20.6	Left	ND	Lunate	2	26	2
13	30/M	16.8	Right	D	Lunate	2	14	3
14	23/F	21.3	Right	D	Lunate	1	48	2
15	25/F	23.6	Right	-	Proximal tibia	1	46	2
16	42/M	19.4	Right	-	Proximal tibia	1	24	1
17	27/F	28.8	Left	-	Metatarsal head	1	36	2
18	32/M	31.2	Right	-	Metatarsal head	1	26	1

### Assessment

At presentation and subsequent follow-ups, patients were assessed clinically for pain, range of movement, and mean analgesic requirement. Visual analogue scale (VAS) was used to assess the intensity of pain and was recorded on a verbal response scale of 0–10 (0 for no pain, 10 for the most severe). Radiological assessment was done with magnetic resonance imaging (MRI) and plain radiographs in anteroposterior and lateral views. AVN for the humeral head, talus, scaphoid, lunate, and metatarsal head was classified as per Cruess [12], modified Ficat and Artlet [13, 14], Herbert and Lanzetta [15], Lichtman et al. [16], and Smillie [17] classification, respectively.

All patients were followed up at 6 weeks, 3 months, every 6 months in the first 2 years, and annually thereafter. At each visit, range of movement and intensity of pain (VAS score) along with mean analgesic requirement were recorded. Radiographs and MRI were taken to note down the radiological improvement in terms of resolution of bone marrow edema and to classify the radiological progression or stabilization of the pathology. Clinical failure was considered when pain and disability warranted surgical intervention. Radiological failure was defined as a progression to arthritis or collapse stage.

### Medical management

Patients received a single injection of intravenous ZA (5 mg) at first visit followed by oral alendronate 70 mg weekly (in divided doses) for 1 year. All patients received oral daily supplements of calcium 500 mg and 400 IU of vitamin D. Analgesics were given as and when required. Partial weight bearing (patients with lower limb AVN) using axillary or elbow crutches was advised for the first 3 months after initiation of therapy, thereafter weight bearing was allowed as tolerated.

### Statistical analysis

Statistical analysis was done using SPSS software Version 20.0 (SPSS Inc., Chicago, IL, USA). Wilcoxon signed-rank test was used to determine the level of significance after confirming the normal distribution of results using the Shapiro-Wilk test. *P* value < 0.05 was considered significant.

## Results

In our analysis of 18 patients with AVNOFH, 5 patients had AVN of the humeral head, 4 patients of the talus, 3 of the lunate, and 2 each of the scaphoid, medial tibial plateau, and second metatarsal head.

### Clinical assessment

Mean VAS pain score reduced from 7.72 at the start of therapy to 3.12 in a mean duration of 4.3 weeks (3–13 weeks) (Table 2). Following this, a gradual decline was observed with mean VAS being 2.44, 0.83, and 0.56 respectively at 1 year, 2 years, and the last follow-up. In accordance with the VAS score, the mean analgesic requirement also dropped significantly. A 50% reduction in mean analgesic requirement was achieved in the first 6 weeks (2–11 weeks) and it remained significantly lower as compared to baseline till the last follow-up. Clinical outcome in terms of range of movement of the corresponding joint also improved significantly in all patients and returned back to the normal limits within 1 year.

**Table 2:** VAS (visual analogue scale) pain score at all follow up visits

Mean VAS	Baseline	4 weeks	1 year	2 year	Last follow-up
Mean (range)	7.72 (5–9)	3.12 (1–6)	2.44 (0–4)	0.83 (0–3)	0.56 (0–3)
*P* value (*t* test)		< 0.0001	< 0.0001	< 0.001	< 0.001

### Radiological assessment

**Fig. 1:**
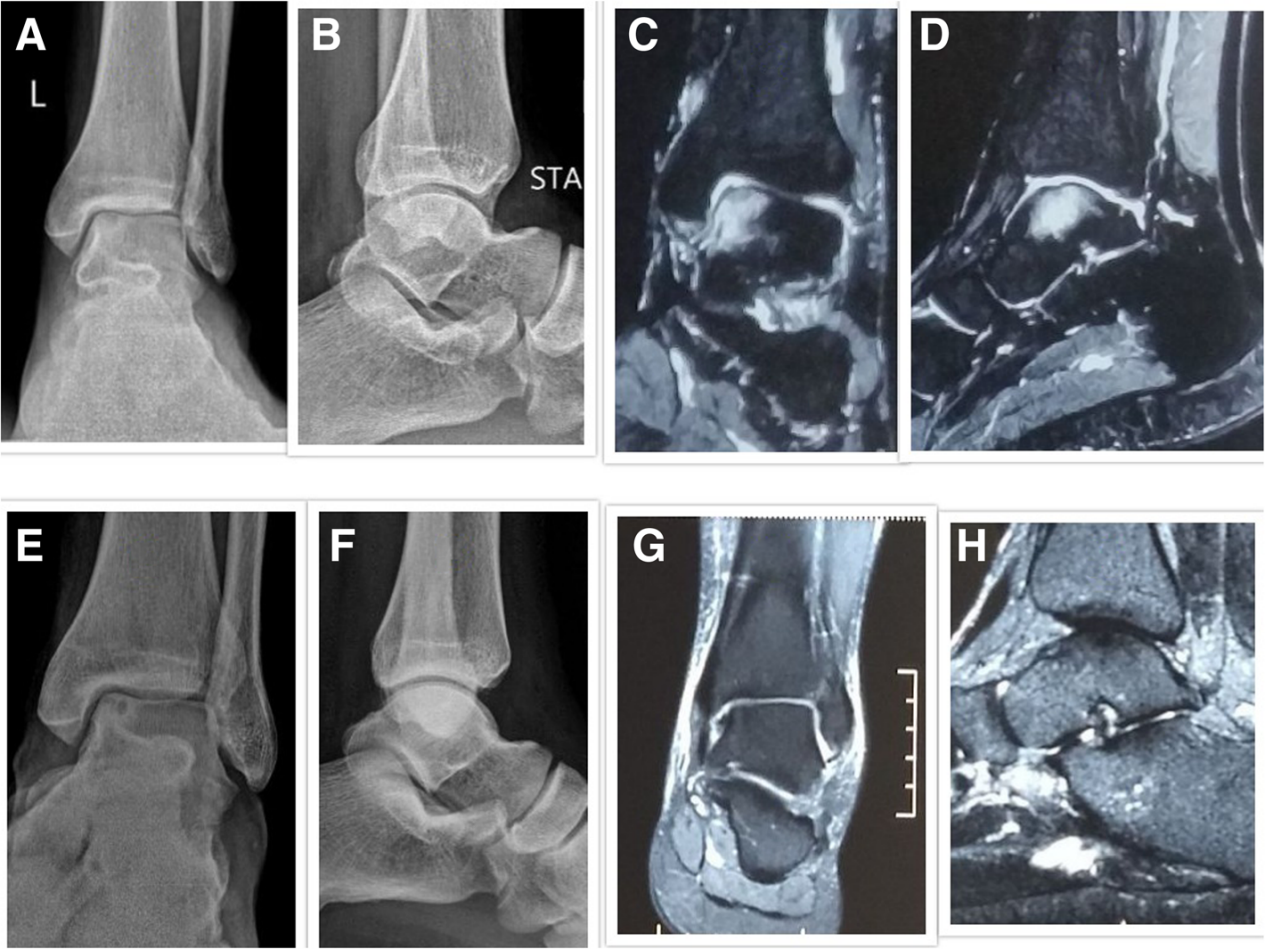
**a**, **b** Pre-treatment anteroposterior and lateral radiographs of a 50-year-old male diagnosed with modified Ficat and Arlet stage II avascular necrosis of the talus bone. **c**, **d** Pre-treatment MRI Coronal and Sagittal images showing BME. **e**, **f** Post-treatment anteroposterior and lateral radiographs at 50 months follow-up showing no radiological collapse/progression with stabilization of the AVN in stage II. **g**, **h** Post-treatment MRI coronal and sagittal images showing complete resolution of BME

**Fig. 2:**
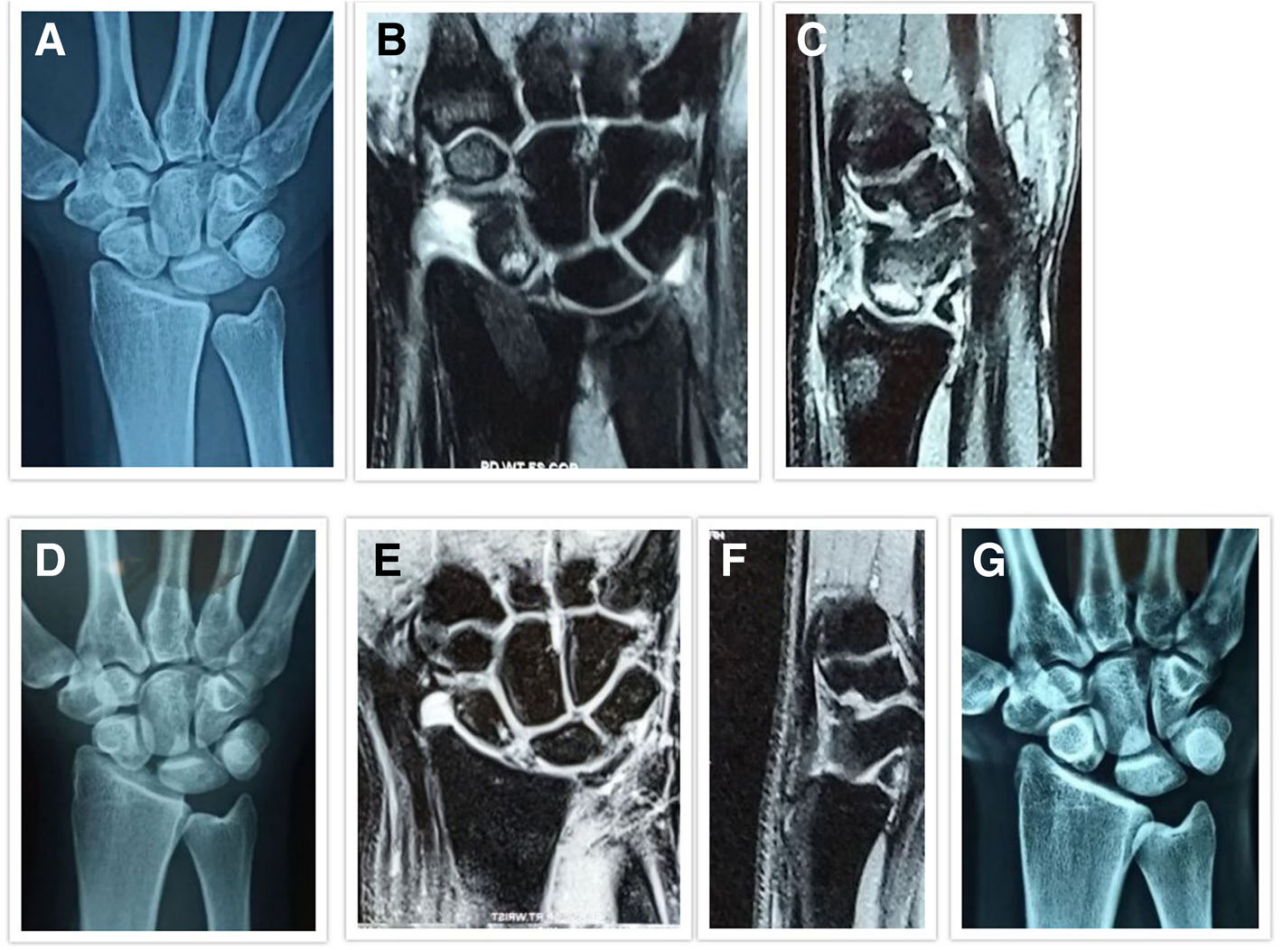
**a** Pre-treatment anteroposterior radiographs of a 23-year-old male diagnosed with Herbert and Lanzetta stage II Avascular necrosis of the scaphoid. **b**, **c** Pre-treatment MRI coronal and sagittal view showing BME. **d** Post-treatment anteroposterior radiographs at 6 months showing no radiological collapse. **e**, **f** Post-treatment MRI coronal and sagittal view at 6 months showing complete resolution of BME. **g** Post-treatment anteroposterior radiographs at 32 months follow-up showing no radiological collapse/progression with stabilization of the AVN in stage II

**Fig. 3:**
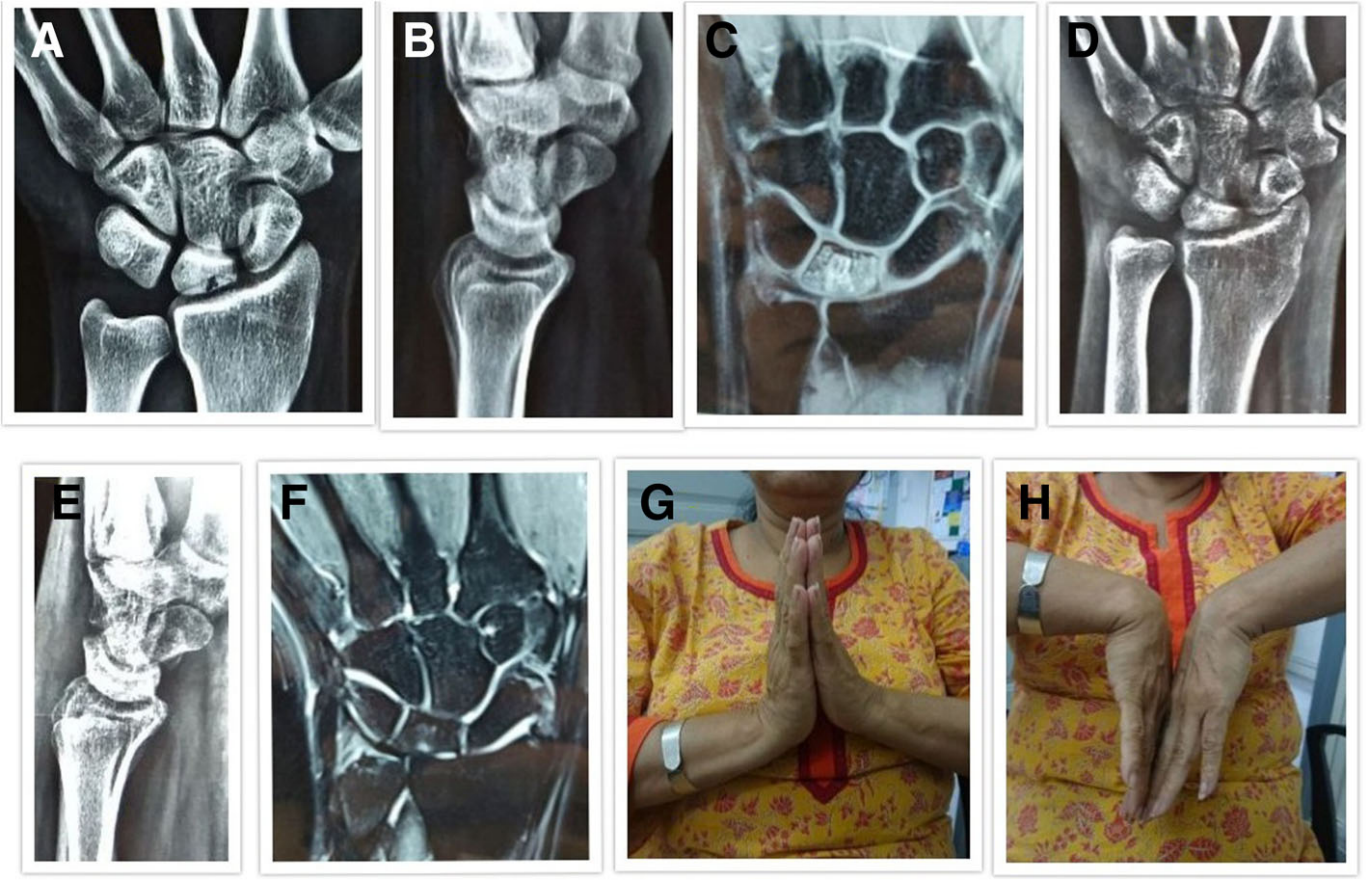
**a**, **b** Pre-treatment anteroposterior and lateral radiographs of a 52-year-old female diagnosed with Lichtman stage II avascular necrosis of the lunate left side. **c** Pre-treatment MRI coronal view of the same patient showing BME. **d**, **e** Post-treatment anteroposterior and lateral views at 26 months follow-up showing no radiological collapse. **f** Post-treatment MRI coronal view showing complete resolution of BME. **g**, **h** Clinical images showing good and comparable range of motion of the left wrist joint as compared to normal right side

At 6 month follow-up, MRI showed complete resolution of BME in 13 patients, > 50% reduction in 4 patients, and less than 50% reduction in 1 patient (Table 3). Seventeen patients (94.4%) had complete resolution of BME at 1 year follow-up. Radiological collapse was seen in 6 out of 18 patients at a mean follow-up of 35.3 months (range 14–56 months) (Table 4). Only one out of 18 patients enrolled required surgery. The patient had AVN of the lunate and had less than 50% reduction of BME at 1 year. Wrist arthrodesis was done at 19 months after the start of therapy. Figures 1, 2, and 3 represent some cases who had non-femoral AVN and were treated with our therapy.

**Table 3:** Table showing the MRI assessment of the patients till last follow-up

MRI	Baseline	6 months	1 year	Last follow-up
Edema present	18 (100%)	5 (27.8%)	1 (5.6%)	1 (5.6%)
Edema absent	0 (0%)	13 (72.2%)	17 (94.4%)	17 (94.4%)

**Table 4:** Table showing the stage-wise proportion of cases underwent radiological progression, collapse, and surgery

Stage at the commencement of treatment	Number of affected joints at presentation	Number of joints had radiological progression	Number of joints had radiological collapse	Number of joints underwent surgery
I	7	4/7 (57.1%)	1/7 (14.3%)	0/7
II	11	5/11 (44.5%)	5/11 (44.5%)	1/11 (9.1%)
Total	18	9/18 (50%)	6/18 (33.3%)	1/18 (5.6%)

There were no major side effects noted necessitating cessation of the therapy in any of the patients. Two patients reported mild gastric upset with the first dose of oral alendronate which subsequently settled down without any intervention and the other doses did not give any symptoms.

## Discussion

### AVN of the humerus

AVN of the humeral head is the second most common of AVN. It is often unrecognized and presents in late stages. It is more common in male and often presents between 20 and 50 years of age. Conservative measures are only meant for pre-collapse stage I and II, while hemiarthroplasty is the treatment of choice for stage III and IV, and total shoulder arthroplasty for stage V [18, 19]. Natural healing is very rare in AVN of the humeral head and most of them progress to the stage of collapse and require surgery [19]. Hernigou et al. reported that 82% of symptomatic patients and 54% of asymptomatic patients with AVN of the humeral head had radiological collapse at their last follow-up [18]. The authors have reported partial or total regression only in asymptomatic stage I AVN. Out of the 5 cases of AVN of the humeral head in our study, 1 was in stage I and 4 were in stage 2. Patient presented with stage I had progressed to stage II at the last follow-up of 48 months. Three patients with Stage II AVN had shown progression to stage III, while 1 case had not shown any radiological progression till the last follow-up of 25 months. None of these patients required surgery after our therapy.

### AVN of the talus

Atraumatic AVN of the talus presents with a treatment dilemma, while there are a number of treatment options available, the outcome remains suboptimal [14]. Treatment methods included are conservative in terms of restricted weight bearing and braces to joint-sparing procedures like bone grafting, core decompression, and joint-sacrificing procedures like talectomy or arthrodesis [13, 20–23]. The most important factor which affects the treatment method choice is the collapse of the talar dome. Conservative treatment is reserved only for early pre-collapse stage; however, most of them eventually progress to the stage of collapse [24]. Joint-sparing procedures can only delay but not stop the progression to collapse and arthritis. Delanois et al. [20] studied 37 cases of atraumatic AVN of the talus, of which 29 cases were in stage II while 8 cases were in stage III. Thirty-five out of 37 cases required some surgical intervention eventually. In our study, 1 of the 3 cases in stage II had progressed to stage III but was asymptomatic. One case with stage I at presentation did progress to stage III at last follow-up of 56 months but did not require surgery.

### AVN of the scaphoid

Atraumatic AVN of the scaphoid is a rare entity and there are no treatment guidelines available till date. Natural history of this disease involves progression to fragmentation and carpal collapse and thus most of them eventually require surgery [15]. Lenoir et al. [4] in a systematic review of 29 articles have shown that conservative treatment in ineffective even in early stages and almost all the cases require surgical treatment. While vascularized bone graft can stop or reverse the disease process in stage II, later stages require either carpectomy or arthrodesis. We have treated 2 cases of AVN of the scaphoid (Stage II) with our combination of oral alendronate and intravenous ZA. At a mean follow-up of 28.5 months, none of them had shown any radiological progression and did not require surgery. Both the patients were asymptomatic and were able to do all activities unaided.

### AVN of the lunate

Treatment of AVN of the lunate (Keinbock’s disease) depends on the stage of presentation. Early stages (Lichtman stage I and II) are usually managed conservatively initially, but most of them eventually progress to late stages (III and IV) [25, 26]. Advanced stage is characterized by carpal collapse, joint incongruity, and osteoarthritis [25]. Management methods in this stage include excision arthroplasty, revascularization procedures, vascular bundle implantation, intercarpal arthrodesis, shortening the radius, or by lengthening the ulna, modified Graner II procedure [25–28]. There is no treatment method available to halt the disease progression in early stages and therefore, most of them eventually require surgery. In our study, out of the three patients presented with AVN of the lunate, 2 patients did not require surgery. One patient who underwent surgery presented with Stage II AVN. He was symptomatic and progressed to Stage III at 14 months. His VAS at the start of the treatment was 6 which did show an initial decline (VAS score of 4 at 3 months) but never decreased thereafter and had a score of 8 at 14 month follow-up, when the patient opted for surgery. Also his mean analgesic requirement also increased over the period of time. Eventually, the patient had to undergo Modified Graner II procedure. He is doing well after the surgery.

### AVN of the metatarsal head

Our study includes 2 patients with AVN of the second metatarsal head/Frieberg disease. Both presented in Smillie stage I AVN and did well with our treatment protocol. They did not require surgery till their last follow-up. Although one of them had progressed to Stage II but was asymptomatic till last follow-up of 36 months. Literature supports that conservative management is reserved only for early stages (I-III) and is aimed at protection of the toe and alleviation of discomfort [29]. Most of the patients who do not respond to conservative management or presented in late stages (III–IV) eventually require surgery [30]. Thus, our treatment protocol has successfully treated patients with early stage AVN of the second metatarsal head in which no treatment method exists.

### AVN of the tibial plateau

AVN of the proximal medial tibial plateau (SPONK; Spontaneous Osteonecrosis of the Knee) is a rare entity. Satku et al. [31] have studied the natural history of SPONK of the proximal tibia plateau with a mean follow-up of 5.6 years. Out of the 21 cases, 2 had acute collapse, 12 had shown progression to osteoarthritis and complete resolution in only 4 cases. Thus, most of the patients with AVN of the proximal medial tibial plateau progress to arthritis and require surgery. We studied 2 cases of AVN of the proximal medial tibial plateau, both of which responded to our treatment method and were asymptomatic at their last follow-up.

This study being a case series has limitations which include lack of a randomized, double-blind prospective study design and lack of a comparison group. A larger series would have been ideal but because of the rarity of the condition with literature reporting case reports only. To our knowledge, this is the largest case series reported till date for the medical management of non-femoral avascular necrosis. Although, on comparison with historical control, this therapy gave earlier relief in pain and shortened the natural history of the disease.

## Conclusion

Out of the 18 patients enrolled in our study, radiological progression to arthritis was seen in only 2 patients at a mean follow-up of 34.3 months (range 14-56 months), while only 1 patient underwent surgery. Thus, this combination of yearly intravenous zolendronic acid and oral alendronate provides a pragmatic solution in the management of AVN other than femoral head. It not only provides pain relief but also prevents long-term radiological progression, thus obviating the need for surgery. 94.4 % of our patients in early stages of AVNOFH showed good clinical improvement. This combination is well tolerated. Thus, we present a new paradigm in the management of a condition lacking standard management guidelines.

## Abbreviations

AVN: Avascular necrosis
AVNOFH: Avascular Necrosis other than femoral head
MRI: Magnetic resonance imaging
VAS: Visual analogue scale
ZA: Zolendronic acid
